# Supplementary material for: Molecular-Charge-Contact-Based Ion-Sensitive Field-Effect Transistor Sensor in Microfluidic System for Protein Sensing
Source: Sensors (Basel). 2019 Aug 2;19(15):3393. doi: 10.3390/s19153393 (PMC6695797; doi:10.3390/s19153393)
Supplement: Supplementary file 1 [file sensors-19-03393-s001.pdf]

Supplementary Materials

Molecular-Charge-Contact-Based Ion-Sensitive Field-Effect Transistor Sensor in Microfluidic System for Protein Sensing

Haoyue Yang and Toshiya Sakata*

Department of Materials Engineering, School of Engineering, The University of Tokyo, 7-3-1 Hongo, Bunkyo-ku, Tokyo 113-8656, Japan

*Corresponding author. E-mail: sakata@biofet.t.u-tokyo.ac.jp

S1. Previous MCC method

Figure S1 shows the change in interfacial potential (ΔV_{out}) obtained by the previously reported MCC method (**Figure 1b**). Upon adding the biotin-coated magnetic beads with streptavidin, ΔV_{out} drastically increased owing to the direct attachment of magnetic beads to the gate insulator and subsequently decreased gradually when the measurement solution flowed. That is, the effect of the direct attachment of magnetic beads to the gate insulator on the electrical responses was very large and then it took a long time to determine the baseline of interfacial potential; therefore, the actual signal used for biomolecular recognition may be distorted in the MCC method shown in **Figure 1b**.

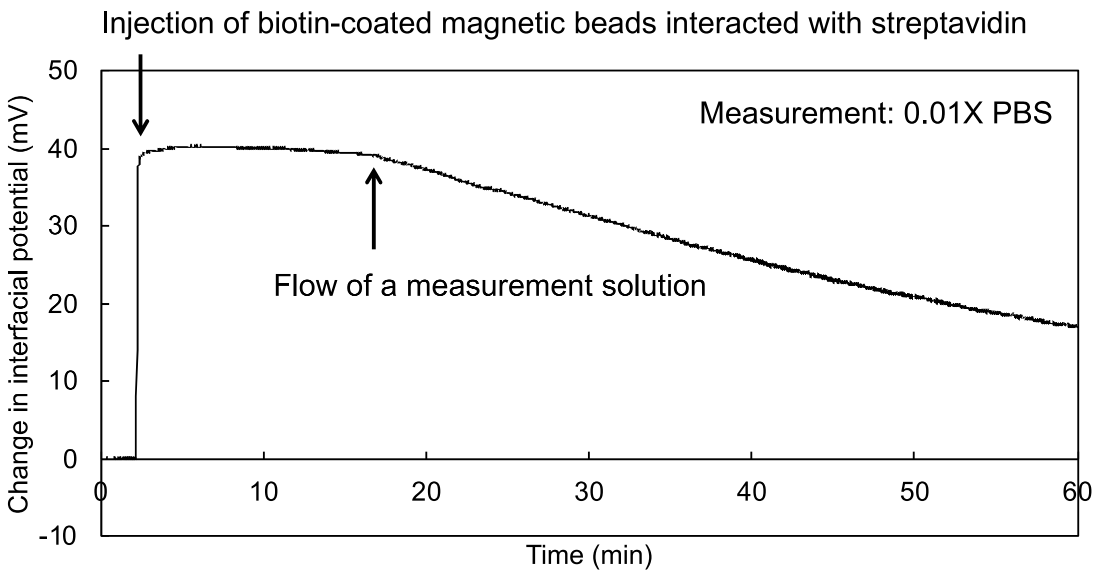

Figure S1. Change in interfacial potential at solution/gate insulator interface for previous MCC method.

S2. Source-follower circuit for FET real-time measurement

In this study, the change in surface potential was monitored using the source-follower circuit shown in **Figure S2** [S1]. Here, V_G and V_D were set to constant values and V_S was controlled at a constant I_{DS}.

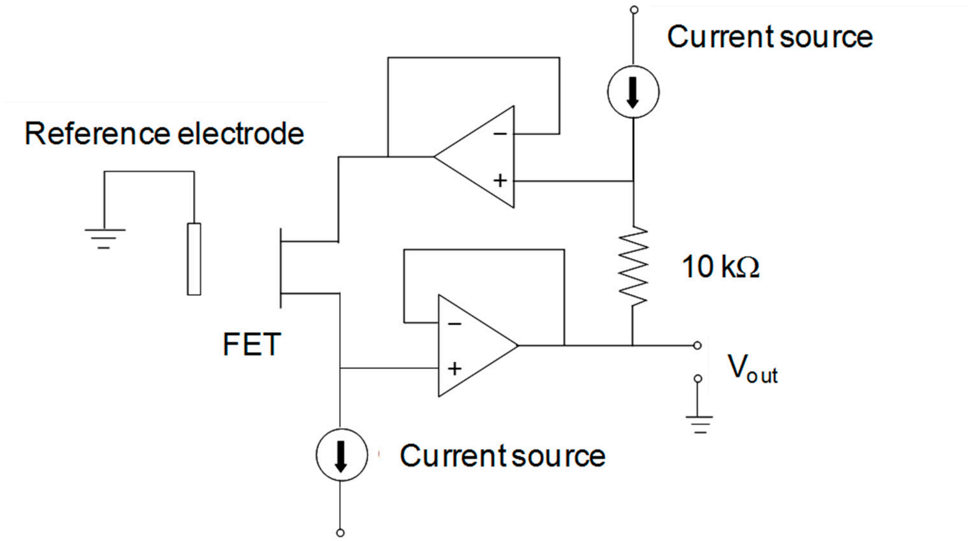

Figure S2. Electrical circuit. The change in surface potential (ΔV_{out}) at the gate was measured at a constant I_D (1 mA) and V_D (2.5 V) using the source follower circuit.

S3. ΔV_2 obtained by the control measurement

Figure S3 shows ΔV_2 obtained by the control measurement without magnetic beads in a variety of PBS solutions. The ionic strength of buffer solution was changed from 1X to 0.01X PBS. ΔV_2 for the ISFET sensor without magnetic beads did not change in the 1X and 0.1X PBS solutions, but shifted in the negative direction by the amount of about 20 mV in the 0.01X PBS solution. This negative shift in ΔV_2 in the 0.01X PBS solution indicated the increase in negative charges at the gate, different from the positive shift in ΔV_2 in the 0.01X PBS solution for the sample measurement (**Figure 4a**). That is, streptavidin molecules with negative charges would have been nonspecifically adsorbed at the gate surface and then detected according to the Debye length limit from the gate surface in the diluted solution. On the other hand, the diluted buffer solutions, such as 0.01X PBS, might have been affected by the dissolution of carbon dioxide in them under ambient conditions.

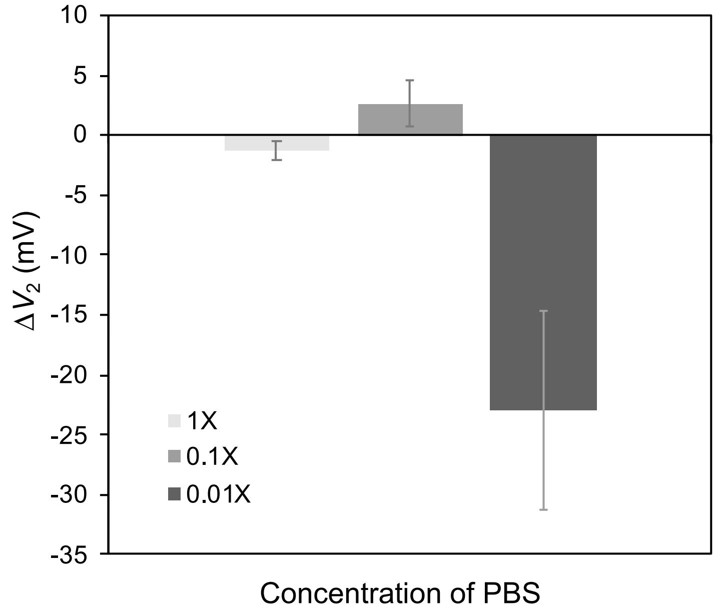

Figure S3. Effect of ionic strength on ΔV_2 in magnetic beads-free measurement.

S4. Calculation of LOD

Considering the Kaiser method, the lowest concentration of streptavidin that can be detected by the MCC method is calculated from ΔV_2^{min} showing a significant difference from the average ΔV_2 at the concentration of streptavidin in the blank ($C = 0$). C indicates the concentration of streptavidin. In **Figure 5**, an approximately straight line is drawn in the concentration range from 1.8 μM to 180 μM, within which streptavidin can be detected, and extrapolated to the ΔV_2 axis. This relationship is expressed as

$$\Delta V_2 = 3.7C + 0.93\ , \qquad (1)$$

where the y-intercept (0.93) means the average ΔV_2 in the blank. Here, the corrected sample standard deviation (σ) is

$$\sigma = \sqrt{\left\{\sum_{i=1}^{n}\left(\Delta V_2^i - \widetilde{\Delta V_2}\right)^2\right\}/(n-1)}\ , \qquad (2)$$

where $\widetilde{\Delta V_2}$ shows the average ΔV_2^i of a detected signal and n indicates the number of detected output signals. Moreover, the reliable range of w in the blank is indicated as

$$w = 3\sigma + 0.93\ . \qquad (3)$$

That is, the LOD (C_{LOD}) is calculated using equations (1)–(3) on the basis of the output signal ΔV_2^{min} (w) that shows $+3\sigma$ from the average ΔV_2 in the blank (0.93).

Considering the above, the LOD for streptavidin for the MCC method was calculated to be about 2.3 μM.

S5. LOD for streptavidin–biotin interaction

Method	Limit of detection (LOD)	Ref.
Potentiometric enzyme immunoassay	80 nM	26
Fluorometric assay	3 ∝M	27
Fluorescence polarization	80 pM	28
Quartz crystal microbalance	300 nM	29
Localized surface plasmon resonance	5 nM	30

Table S1 Limit of detection (LOD) for streptavidin–biotin interaction detection reported in previous papers.[26-30] These references are shown in the article.

S6. Electrical characteristic of ISFET sensor

Figure S4a shows the concept of ISFET sensor. The gate (Ta_2O_5/SiO_2) of the ISFET was set in the polycarbonate ring with a diameter of 18 mm (1 mL). That is, the change in pH in the polycarbonate ring, where a buffer solution was poured, was continuously monitored using the ISFET sensor. The standard buffer solutions (pH 4.01, 6.86, 7.41, and 9.18) were used for evaluating pH responsivities. The semiconducting material is separated from the solution by the gate insulator, the thickness of which is in the order of 100 nm. The gate insulator is often composed of an oxide, such as SiO_2, Ta_2O_5, Al_2O_3 or nitrides (e.g., Si_3N_4) [S2]. The hydroxy groups are formed at the surface of the gate insulator in the solution and are thus responsive to hydrogen ions (**Figure S4a**). These positive charges at the gate surface electrostatically interact with electrons at the channel in the silicon substrate. The field effect induced by the changes in the charge densities at the gate causes a change in the threshold voltage (ΔV_T) at a constant drain source current (I_D) in the gate voltage (V_G)–I_D electrical characteristic. This electrical response of the ISFET to hydrogen ions is Nernstian at about 59.1 mV/pH at 25 °C [S3,S4]. In fact, ΔV_T was measured with changes in pH using one of the ISFET sensors in this study (**Figure S4b**). From such electrical characteristics, the average pH sensitivity was found to be about 55 mV/pH at 25 °C for the 6 ISFET sensors used in this study, as shown in **Figure S5c**. According to the detection principle, the surface potential at the gate surface was continuously monitored using a source follower circuit, as shown in **Figure 3**. As a result, the potential change (ΔV_{out}) at the interface between the gate insulator and an aqueous solution, which corresponds to -ΔV_T, can be directly output at a constant I_D [S1].

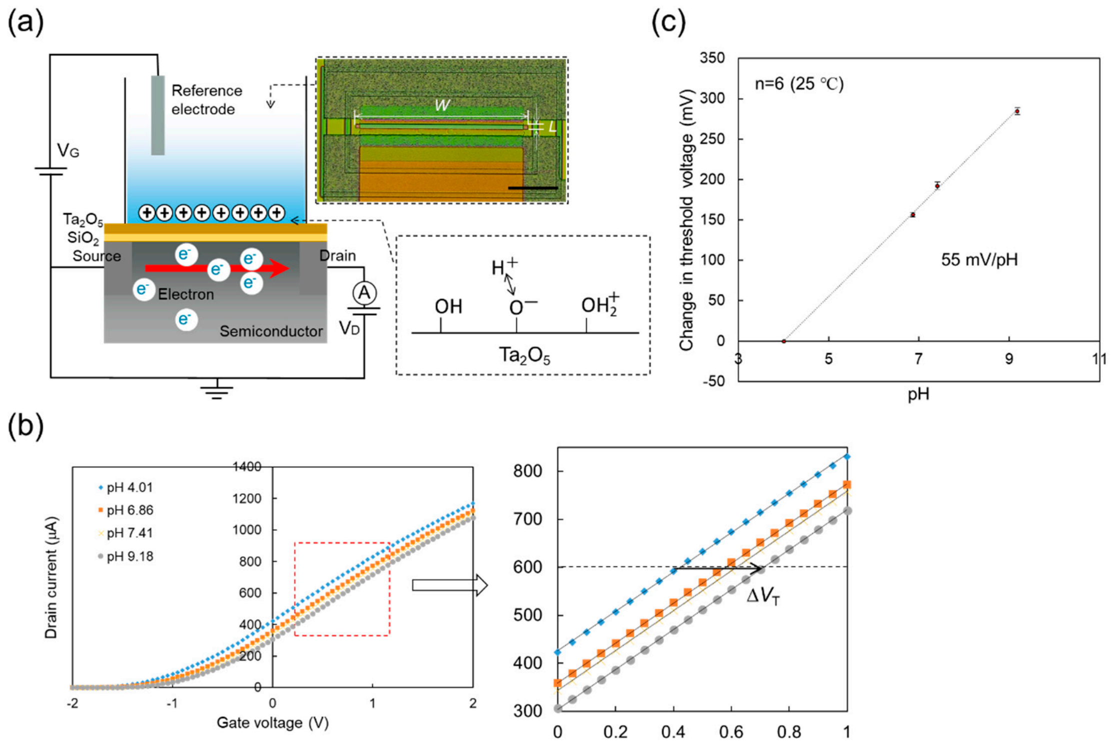

Figure S4. (**a**) Conceptual structure of ISFET sensor for electrical measurements. The channel was designed to have a width (W) and length (L) of 340 and 10 µm, respectively. Scale bar = 100 µm. Hydroxy groups at the oxide membrane in a solution exhibit the equilibrium reaction with hydrogen ions. (**b**) V_G–I_D electrical characteristic of one of the ISFET sensors used in this study. The shift in V_G at a constant I_D of 1 mA was estimated as the change in V_T when the pH was changed from a pH of 4.01 to 9.18. (**c**) Calibration curve, which was analyzed based on the data shown in (b). The pH sensitivity of this ISFET sensor was about 55 mV/pH, which almost showed a Nernstian response at 25 °C. V_G at a pH of 4.01 was offset to 0.

REFERENCES

S1. Sakata, T., Kamahori, M., and Miyahara, Y. (2005). DNA analysis chip based on field effect transistors. Jpn. J. Appl. Phys. *44 (No. 4B)*, 2854–2859.

S2. McBride, P.T.; Janata, J.; Comte, P.A.; Moss, S.D.; Johnson, C.C. Ion-selective field effect transistors with polymeric membranes. *Anal. Chim. Acta* **1979**, *108*, 239–245.

S3. Bergveld, P. Development, Operation and Application of the Tool for Electrophysiology. *IEEE Trans. Biomed. Eng.* **1972**, *BME-19*, 342−351.

S4. Esashi, M.; Matsuo, T. Integrated micro-multi-ion sensor using field effect of semiconductor. *IEEE Trans. Biomed. Eng.* **1978**, *BME-25*, 184−192.
